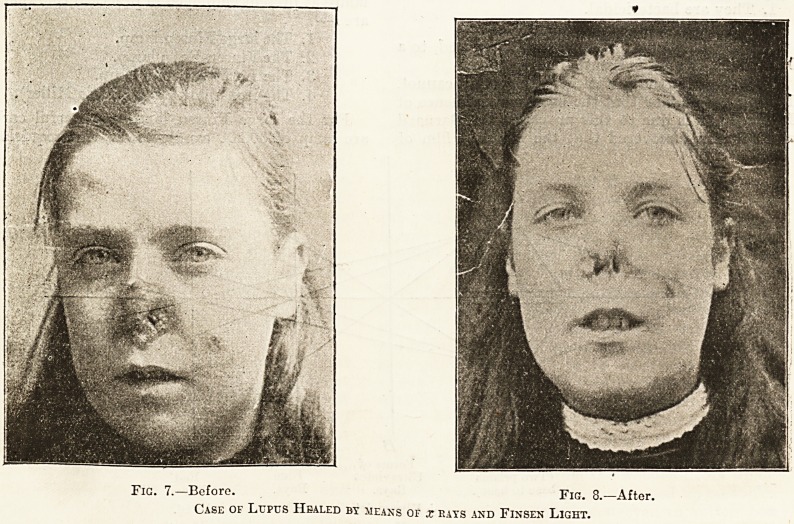# The Present Position of Radiation in Treatment

**Published:** 1906-06-09

**Authors:** Gerald Sichel

**Affiliations:** Surgeon-in-charge of the Actino-therapeutic Department, Guy's Hospital.


### Hospital Clinics.

### THE PRESENT POSITION OF RADIATION IN TREATMENT.

By Gerald Sichel, F.R.C.S., F.C.S., Surgeon-in-charge of the Actino-therapeutic Department, Guy's Hospital.

### II.—Finsen Light.

Finsen's phototherapy utilises the blue, indigo, and violet rays of the visible spectrum, together with the invisible ultraviolet rays just beyond. Barnard and Morgan have located the bactericidal influence to the central portion of the ultraviolet rays.

The essential properties of the Finsen light rays may be tabulated as follows:—

1. They are bactericidal.
2. They have a powerful chemical action.
3. Glass is opaque; pure rock salt, ice, and, to a less extent, quartz, is transparent to them.

With regard to the bactericidal property, it cannot, I think, be claimed that the beneficial influence of Finsen light is due to this property, for Barnard and Morgan discovered that the slightest film of organic matter—such as even a very thin layer of agar—rendered this power inoperative.

Even a superficial patch of non-ulcerating lupus is, of course, covered by an organic layer, which comes between the tubercle bacilli and the rays; therefore the curative power of the light is probably due, not to the bactericidal, but to the chemical rays, causing stimulation and increased phagocytosis.

The third and final fact tabulated above is important, since it much increases the expense of the apparatus, as it is, of course, impossible to use glass lenses; they must be made of quartz, or else ice be used.

It has been found that, in order to render the treatment efficient, the part to be treated must be rendered practically bloodless during the exposure by means of pressure.

Another essential point to remember is that Finsen light is a concentrated light, from which the heat rays are excluded by filtering through water; and it is a practical point very well worth recollecting among those first using the apparatus that the first duty of the nurse or other operator is to see that the water circulation is in full working order before the electric current is switched on. Finsen's own opinion was that the sun was the best source of the rays, but for various and obvious reasons the arc electric light is most conveniently used. The lamps at present employed in practical therapeutics are:—

1. The large Finsen lamp.
2. The Finsen-Reyn lamp.
3. The Lortet-Genoud lamp.
4. The iron-spark lamp (Leslie Miller).

For the large Finsen lamp a powerful carbon arc lamp of 30,000 candle-power, and requiring a current of 60 ampères, is the source of light. Around this light are arranged four water-cooled "telescopes" to focus and thus concentrate the light on the patient.

The heat rays are eliminated by means of filtering through distilled water contained in the "telescopes," and the Finsen rays being refracted more than the rest of the spectrum when passed through a prism, it is well to keep the spot to be treated just inside the actual visible point of focus. The following diagram will make this point clear.

Fig. 6.

Finally, the part to be treated is rendered anæmic by pressure with a "compressor," consisting of two quartz plates separated by a small chamber cooled by a constant flow of water.

The treatment is conducted by a trained nurse, and each sitting lasts one hour.

If the treatment, which is painless, except in exceptional cases, has been properly carried out, the symptoms of sunburn appear, and within two days a blister may make its appearance. To prevent pus-inoculation unguentum hydrargyri oxidi flavi should be applied, and in a week or ten days the part can be treated again.

Another important point to remember in the treatment is that the end of the telescope and the part to be treated should be exactly parallel to each other. Altogether, it may be said that the Finsen light treatment depends for its success on extremely careful attention to minutiæ, and it therefore requires the supervision of the specialist, and not the casual operator. Indeed, there are a hundred and one points of special importance which call for notice, but would be out of place in a paper of this character.

The Finsen-Reyn lamp is in principle exactly the same as the large Finsen lamp, except that, instead of four, only one patient is treated at a time; and as it requires a current of 20 ampères, it is a little more expensive to run per patient. However, in certain cases it is more suitable.

The Lortet-Genoud lamp requires no separate compressor, as the patient simply presses the afflicted part against the lens of the lamp.

The iron-spark lamp has the great advantage of producing a reaction in much shorter time than the others, which are all carbon arc lamps. Another advantage is that practically no heat is evolved, and therefore no expensive water-cooling apparatus is necessary. Either a quartz plate, or, better still, a piece of ice (as suggested by Dr. Walsham, of Bartholomew's Hospital), is used for pressure to render the part anæmic, and the sittings last ten or fifteen minutes.

It is, in my opinion, the lupus lamp for the general practitioner, as it is worked from an ordinary $x$-ray coil through a condenser; but care must be taken to choose a reliable design. I was grievously disappointed with the first one I tried. The one we now use, made by Leslie-Miller, gives excellent results. At St. Bartholomew's it is the only form of lamp used for lupus by Dr. Lewis Jones. Where, however, expense is no object, my experience is that the best results are obtained with the large Finsen lamp.

The chief difficulty with the Finsen lamp at Guy's has been with the carbons, for, unless the proper kind and size are obtained, they are apt to flame and burn unequally. At Guy's we are getting very good results with Siemens' A quality cadmium core carbons, size 30 m.m. for the positive, and size 24 m.m. for the negative.

Fig. 7.—Before.

Fig. 8.—After.

Case of Lupus Healed by means of $x$ rays and Finsen Light.

The ordinary light bath, supplied with incandescent lamps, produces but a poor amount of chemical rays, but is rich in red, yellow, and green rays. In other words, perhaps "radiant heat bath" is a better term than light bath, except for the fact that very many workers are disposed to think that the red end of the spectrum may quite likely be found to have quite as useful a field in therapeutics as the violet, quite apart from the temperature effects.

At the present time there are very few who would deny Finsen light the first place in the treatment of lupus. If there still be any disbelievers, I would recommend such to visit the London Hospital and get Dr. Sequeira to show them his photographs.

In cases of lupus there are many which, in the first place, are ulcerated and too tender to stand the pressure required to render them anæmic. Such spots are far better treated with $x$-rays. Those cases, again, in which there is much induration and thickening, do better if at first treated with $x$-rays; but when the ulceration or induration has disappeared, then the application of Finsen light gives the best results. There are a few cases, however, in which even Finsen light is unable to bring about healing; these are the cases in which, I think, serotherapy might with benefit combine with phototherapy.

In a general hospital there is often a certain amount of difficulty quite naturally in arranging for a dual treatment for the patient, and at present the number of cases available for statistics are far too few to draw any reliable data from.

Phototherapy has been applied successfully in cases of alopecia areata by Continental observers. I am at present treating three cases of universal alopecia with diffuse electric carbon arc light: two are congenital, and at present show no sign of improvement; the third, an acquired case, is beginning to get a down-like crop of very fine light hairs. It is only fair to add that it was at the suggestion of the sister of the department that the treatment is being tried.

With regard to lupus erythematosus, I think it is best not to attempt treatment with photo- (or, indeed, any other form of radio-therapy), as it is apt to make the condition worse. Finsen light has been used in a variety of other skin affections with, so far as I am aware, little or no success; but if its field of usefulness seems limited to practically only one disease (lupus), still the sufferers are so numerous, the disease so terrible, and the results of its treatment so satisfactory, Finsen light must still be looked upon as an extremely valuable addition to the surgeon's armamentarium.

Considering also the great benefit received by neurotic patients in many cases of exposure to fresh air and sunlight, it seems to me possible that this class of patient, the despair alike of themselves, their friends, and the physician, might reasonably be expected to do well with light treatment; and a perfectly legitimate and safe line of experimental therapeutics might determine which rays, if any, had an influence for the good.

## Figures and Tables

**Fig. 6. f1:**
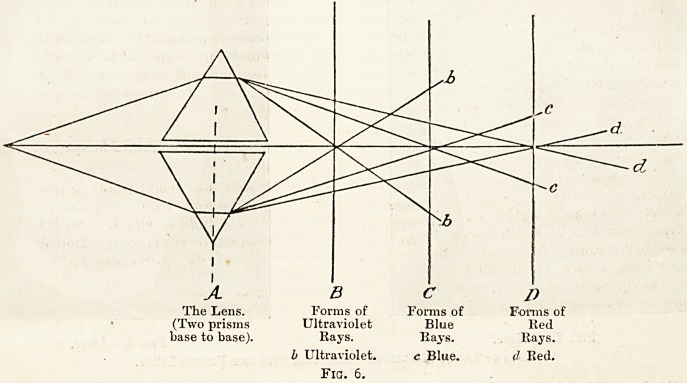


**Fig. 7. Fig. 8. f2:**